# Pulsed field ablation of incessant superior vena cava–triggered atrial fibrillation: watch out for the sinoatrial node

**DOI:** 10.1007/s10840-023-01624-7

**Published:** 2023-08-17

**Authors:** Shaojie Chen, David Schaack, Jun Hirokami, Boris Schmidt, Julian K. R. Chun

**Affiliations:** 1 Cardioangiologisches Centrum Bethanien (CCB), Kardiologie, Medizinische Klinik III, Agaplesion Markus Krankenhaus, Akademisches Lehrkrankenhaus der Goethe-Universität Frankfurt am Main, Frankfurt am Main, Germany; 2 Die Sektion Medizin, Universität zu Lübeck, Lübeck, Germany

Pulsed field ablation (PFA) represents a safe and efficient ablation technology [1–4]. A 60-year-old female patient (BMI 22 kg/m^2^) had highly symptomatic paroxysmal atrial fibrillation (AF) for 1 year. The patient reported that the AF episode had become significantly more frequent in the last 1 month. She had a CHADS-VASc score of 1. Echocardiography showed no pathologic finding, normal-sized heart, left atrium diameter (39 mm), and left ventricular ejection fraction (LVEF) of 72%. She had known asymptomatic sinus bradycardia and received no antiarrhythmic drug treatment.

The patient underwent PFA. Despite pulmonary vein isolation (PVI) and cardioversions, there were incessant spontaneous atrial activities which eventually developed into sustained AF (Fig. 1).

**Fig. 1:**
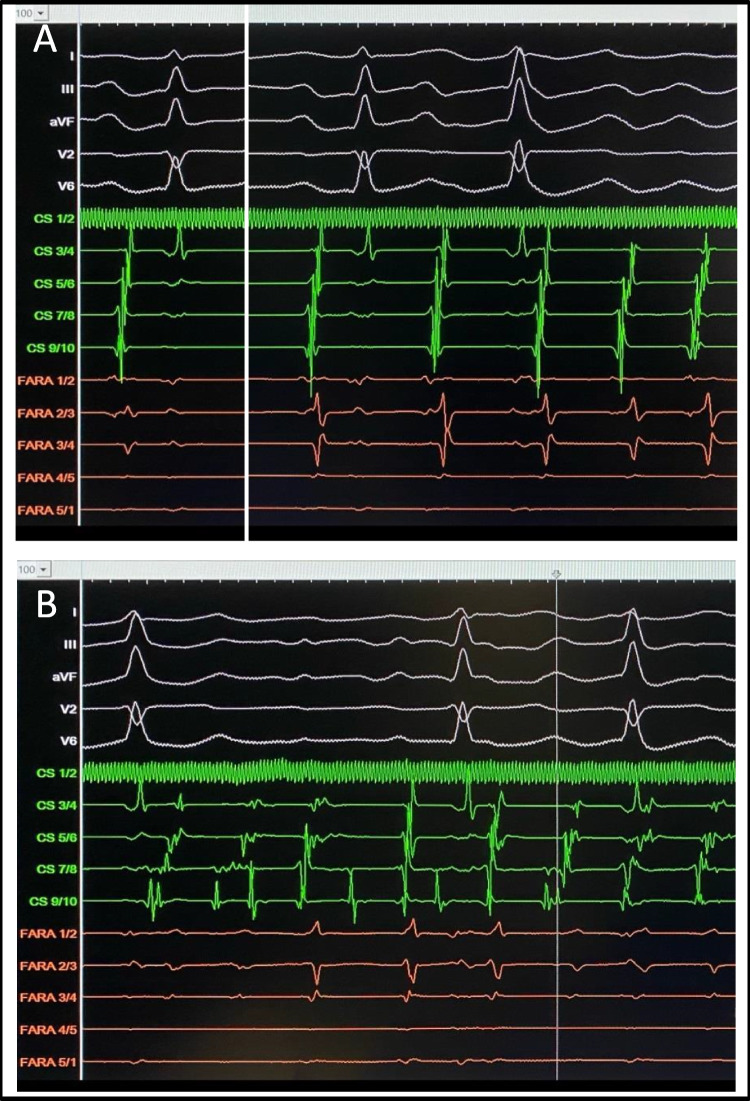
Despite successful pulsed field ablation (PFA)-based PVI, we observed **A** reproducible spontaneous initiation of atrial activities with early activation at the proximal coronary sinus (CS) and late FARAWAVE activation in the left atrium (LA). **B** These atrial activities eventually developed into AF which can be then spontaneously terminated. Such incessant “atrial firing → AF → termination → atrial firing → AF → termination” cycle gave us a chance to map the origin of the trigger

The atrial “firing” was mapped, and it showed the earliest activation from the superior vena cava (SVC) (Fig. 2A). The SVC was successfully isolated by one PFA (FARAWAVE 31 mm, 2.0 kv) application, resulting in the elimination of the atrial “firing” (Fig. 2B–D). Thereafter, the patient had sustained an escape rhythm, and it lasted even after completely withdrawing the sedation (propofol), but the sinus rhythm finally recovered after 30 min (Fig. 3). In the hospital, continuous monitoring showed stable sinus rhythm. Six-month follow-up showed no clinical recurrence.

**Fig. 2:**
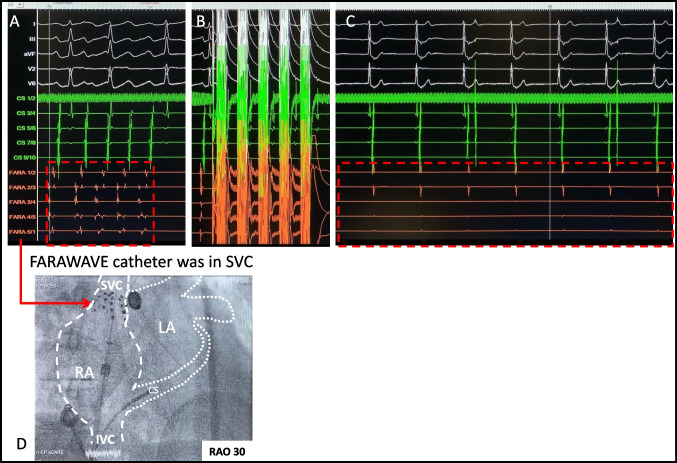
**A** Electrogram: FARAWAVE PFA catheter was positioned at RA-SVC junction, reproducible observation of earliest firing from SVC with identical CS activation and P wave as previously shown in Fig. 1. **B** 1st PFA application. **C** After 1st PFA, visualized elimination of SVC potential, no more SVC firing, sustained escape rhythm HR~40/min. **D** Fluoroscopic position of the FARAWAVE PFA catheter

**Fig. 3:**
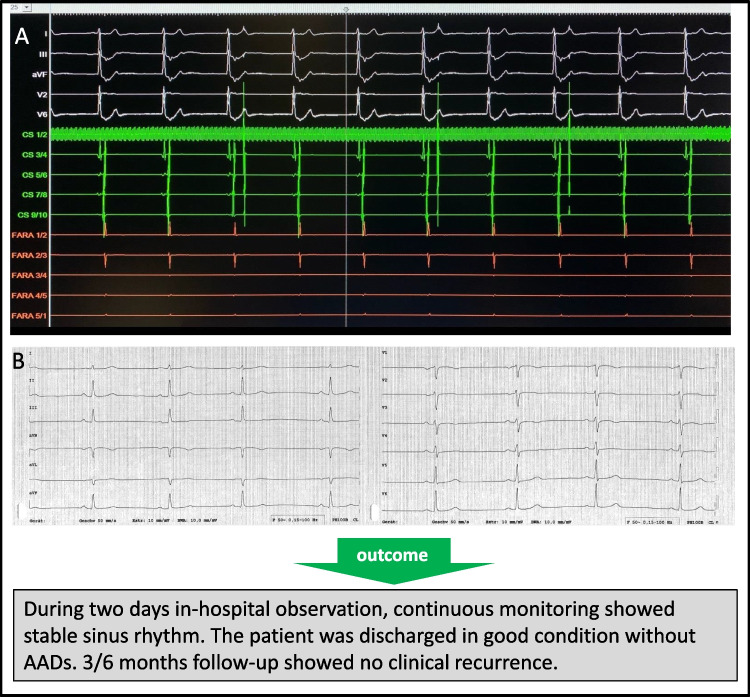
**A** After 1st PFA application, procedural observing: no more SVC firing, and sustained escape rhythm HR~40/min, even after withdrawing the sedation. **B** 30 min later, sinus rhythm recovered with HR 50–60/min (note: ECG speed 50 mm/s, the patient had known sinus bradycardia)

## Discussion

There are maybe several learning points from this case report. Based on our observation (center > 10,000 AF procedures), intra-procedural incessant SVC-triggered AF seems seldom in the European population. This case reported intra-procedural incessant SVC-triggered AF despite PVI, and PFA readily isolated the SVC and eliminated the SVC trigger even with one application.

The mechanisms of the sinus arrest and escape rhythm after the PFA application at SVC need further discussion and investigation. Based on our fluoroscopy-guided catheter manipulation and local electrogram, the PFA application was performed in basket configuration at the right atrium (RA)-superior vena cava (SVC) junction. SVC isolation using PFA resulting in sinus arrest has been reported in a preclinical study. Yavin et al. conducted a preclinical study in a swine model to investigate the expandable 34-mm lattice-tip catheter (SpherePVI™, Affera Inc.) for single-shot PVI with PFA. In their study, the SVC and right superior pulmonary vein (RSPV) were targeted for isolation. In particular, they found one case where PFA for SVC isolation resulted in sinus node arrest and junctional escape rhythm [5].

However, in our case, one limitation is that we did not routinely use ICE (intracardiac echocardiography) or 3D mapping to visualize accurate catheter position during ablation.

We also thought about the autonomic response as a possible mechanism for the sinus arrest and escape rhythm, for there are known ganglion plexus at the RA-SVC junction. The incidence of autonomic response (e.g., sinus arrest, AV block III) during PFA for PVI was not that seldom; however, based on our (> 600 PFA) experience, we have not observed such long (30 min) recovery time from an autonomic response in previous PFA-based PVI cases.

In our center, we do not conventionally perform SVC isolation unless clearly reproducible triggered AF during the procedure. This case was actually the first case of SVC isolation using PFA, and our observation needs further investigation.

The patient had a known history of sinus bradycardia. During the procedure, the patient was carefully sedated under continuous infusion of propofol (1%). The sustained escape rhythm lasted even after completely withdrawing the sedation. We compared the ECG and vital parameters before and during sedation, and no relevant difference was observed. These suggested that the sedation (propofol) may not play a significant role for the sinus arrest and escape rhythm in this case.

Nonetheless, due to the anatomic proximity of the SCV ostium and the SAN, PFA at the RA-SVC junction near SAN poses a risk of SAN dysfunction; therefore, PFA for SVC isolation more at the SVC side instead of at the atrial side is advisable.
